# Combination of Rescue Stenting and Antiplatelet Infusion Improved Outcomes for Acute Intracranial Atherosclerosis-Related Large-Vessel Occlusion

**DOI:** 10.3389/fneur.2021.608270

**Published:** 2021-07-05

**Authors:** Jang-Hyun Baek, Cheolkyu Jung, Byung Moon Kim, Ji Hoe Heo, Dong Joon Kim, Hyo Suk Nam, Young Dae Kim, Eun Hyun Lim, Jun-Hwee Kim, Jun Yup Kim, Jae Hyoung Kim

**Affiliations:** ^1^Department of Neurology, Kangbuk Samsung Hospital, Sungkyunkwan University School of Medicine, Seoul, South Korea; ^2^Department of Neurology, Severance Stroke Center, Severance Hospital, Yonsei University College of Medicine, Seoul, South Korea; ^3^Department of Radiology, Seoul National University Bundang Hospital, Seongnam, South Korea; ^4^Interventional Neuroradiology, Department of Radiology, Severance Hospital, Severance Stroke Center, Yonsei University College of Medicine, Seoul, South Korea; ^5^Department of Neurosurgery, Andong Hospital, Andong, South Korea; ^6^Department of Neurology and Cerebrovascular Center, Seoul National University Bundang Hospital, Seongnam, South Korea

**Keywords:** stroke, endovascular treatment, atherosclerosis, stent, angioplasty

## Abstract

**Background and Purpose:** Intracranial atherosclerosis-related large-vessel occlusion caused by *in situ* thrombo-occlusion (ICAS-LVO) has been regarded an important reason for refractoriness to mechanical thrombectomy (MT). To achieve better outcomes for ICAS-LVO, different endovascular strategies should be explored. We aimed to investigate an optimal endovascular strategy for ICAS-LVO.

**Methods:** We retrospectively reviewed three prospective registries of acute stroke underwent endovascular treatment. Among them, patients with ICAS-LVO were assigned to four groups based on their endovascular strategy: (1) *MT alone*, (2) rescue intracranial stenting after MT failure (*MT-RS*), (3) glycoprotein IIb/IIIa inhibitor infusion after MT failure (*MT-GPI*), and (4) a combination of MT-RS and MT-GPI (*MT-RS*+*GPI*). Baseline characteristics and outcomes were compared among the groups. To evaluate whether the endovascular strategy resulted in favorable outcome, multivariable analysis was also performed.

**Results:** A total of 184 patients with ICAS-LVO were included. Twenty-four patients (13.0%) were treated with MT alone, 25 (13.6%) with MT-RS, 84 (45.7%) with MT-GPI, and 51 (27.7%) with MT-RS+GPI. The MT-RS+GPI group showed the highest recanalization efficiency (98.0%). Frequency of patent arteries on follow-up (98.0%, *p* < 0.001) and favorable outcome (84.3%, *p* < 0.001) were higher in the MT-RS+GPI group than other groups. The MT-RS+GPI strategy remained an independent factor for favorable outcome (odds ratio, 20.4; 95% confidence interval, 1.97–211.4; *p* = 0.012).

**Conclusion:** Endovascular strategy was significantly associated with procedural and clinical outcomes in acute stroke by ICAS-LVO. A combination of RS and GPI infusion might be an optimal rescue modality when frontline MT fails.

## Introduction

Intracranial atherosclerosis-related large-vessel occlusion (ICAS-LVO) caused by *in situ* thrombo-occlusion is a common etiology in endovascular treatment (EVT) for acute stroke. ICAS-LVO was frequently reported in ~17–30% of patients who underwent EVT in Asia, although the incidence varied, depending on race or occlusion site (1). Importantly, mechanical thrombectomy (MT) techniques, including stent retriever thrombectomy (SRT) and contact aspiration thrombectomy (CAT), are ineffective in EVT for acute stroke primarily caused by ICAS-LVO (2–5). Owing to the refractoriness, the number of device passes can be increased in ICAS-LVO, which could delay time to recanalization and also make patient's prognosis worse (6, 7). Furthermore, arterial injury can be possible when stent retriever is indiscriminately applied to ICAS-LVO (8). Stent retriever is also likely to provoke platelet activation on atheromatous plaque, leading to reocclusion of partially recanalized artery (3).

To overcome occlusions refractory to treatment, specific rescue treatments appropriate for ICAS [ICAS-specific modalities (ISMs)], including intracranial stenting, balloon angioplasty, and glycoprotein IIb/IIIa inhibitor (GPI) infusion, can be considered (9–13). In several previous studies, ISMs were associated with higher possibility of successful recanalization, shorter time to recanalization, and better patient outcome (14–18). Although the necessity and feasibility of ISMs have been widely recognized, a practical endovascular strategy for ICAS-LVO has not yet been established. Reliable information regarding which ISM is optimal when the frontline MT fails is lacking. For example, GPI is an efficient rescue modality for the reocclusive ICAS-LVO; however, we do not know exactly when we need a more aggressive modality such as emergent intracranial stenting after GPI injection. While intracranial stenting may ultimately offer a successful recanalization, it might not always be the case. If further intracranial stenting cannot guarantee a successful recanalization, one may have to reconsider the type of ISMs. To determine a practical endovascular strategy for ICAS-LVO, procedural and clinical benefits of each ISM for MT failure should be investigated. Accordingly, the procedural efficiencies and clinical outcomes were evaluated in the present study based on endovascular modality for treatment of acute ICAS-LVO.

## Methods

### Participants

Consecutive acute stroke patients with an intracranial LVO of anterior circulation, who underwent EVT between January 2010 and December 2018 in three comprehensive stroke centers, were retrospectively reviewed. The intracranial internal carotid artery and M1 segment of middle cerebral artery were defined as intracranial large vessels. In the present study, patients who met the following criteria were selected from the prospective registry: (1) first endovascular modality was MT (SRT and/or CAT); (2) age ≥18 years; (3) initial National Institutes of Health Stroke Scale (NIHSS) score ≥4; (4) presentation to the hospital within 8 h from stroke onset; patients within 8–12 h were also considered if they had an Alberta Stroke Program Early CT Score (ASPECTS) ≥7; and (5) premorbid modified Rankin Scale (mRS) score ≤2. Patients whose occlusion etiology was ICAS-LVO were finally included in this study. ICAS-LVO was determined angiographically. Residual fixed focal stenosis >70% of the target vessel or occlusion at arterial trunk on digital subtraction angiography was defined as ICAS-LVO (12, 14). ICAS-LVO was assessed by two independent neurointerventionalists. The κ-value for the interrater reliability was 0.91. Discrepant cases were resolved by consensus.

### Endovascular Procedure

All endovascular procedures were performed under local anesthesia. Conscious sedation was allowed as necessary. The MT procedures were performed according to common recommendations and previous reports (9, 19). Rescue treatments were performed when occlusion was refractory even after several attempts using the frontline MT device; the occlusion segment was recanalized with severe stenosis leading to significant flow limitation, or the occlusion tended to reocclude. Rescue endovascular modalities included switching to the other MT modality (SRT to CAT or *vice versa*), a combination of SRT and CAT, intra-arterial urokinase infusion, balloon angioplasty, intracranial stenting, and/or intra-arterial or intravenous GPI infusion. Selection of the optimal rescue modality depended on the operator's judgment. However, when ICAS was suspected as the cause of LVO, the patient was treated by one of the following endovascular modalities: (1) intracranial rescue stenting with or without balloon angioplasty (RS), (2) GPI infusion, or (3) both modalities. Typically, 5–10 mg of abciximab (1–2 mg/min) or 0.3–1.5 mg of tirofiban (0.05 mg/mL concentration with 0.1 mg/min) was used. A sequence of RS and GPI infusion was not specified. For RS, Solitaire® (Medtronic, Dublin, Ireland) or Wingspan® (Stryker, Kalamazoo, MI, USA) was used. To secure the stability of arterial patency achieved using RS and/or GPI infusion, serial delay angiograms were taken for at least 20 min after recanalization was achieved. When significant angiographic worsening in arterial patency and perfusion were not observed, the procedure was finished.

Successful recanalization was defined as achieving modified TICI grade 2b or 3 and no reocclusion observed on delayed angiograms during the procedure. Dichotomized modified TICI grades (0–2a vs. 2b−3) were assessed by two independent neurointerventionalists blinded to clinical information and follow-up imaging. The κ-value for the interrater reliability was 0.81. All discrepant cases were resolved by consensus.

### Postprocedural Antithrombotic Medication and Follow-Up Examinations

The types and timing of postprocedural antithrombotic medication were determined by consensus of neurointerventionalists and stroke neurologists from each participating center. Although not regulated under specific protocols, the postprocedural antithrombotic medication was summarized as follows: (1) no antithrombotic medication until intracranial hemorrhage was excluded based on brain imaging on the next day of EVT, (2) administration of single (aspirin 100–300 mg or clopidogrel 75 mg) or dual oral antiplatelets (aspirin 100–300 mg with clopidogrel 75 mg) immediately after completion of the EVT procedure, and (3) intravenous infusion of GPI after completion of the EVT procedure for at least 12 h, then administration of dual antiplatelets after exclusion of intracranial hemorrhage based on brain imaging on the next day of EVT.

Arterial patency of recanalized arteries was evaluated on follow-up magnetic resonance angiograms (MRAs) and routinely performed at 1–7 days after EVT. For some patients who were medically unstable, CTA was obtained instead of MRA. The artery was considered patent when significant distal flow was observed on time-of-flight MRA. The arterial patency on follow-up was assessed by two independent physicians who were blinded to final recanalization status and clinical symptoms. The κ-value for the interrater reliability was 0.89. Discrepant cases were resolved by consensus.

### Clinical Variables

All clinical parameters, including functional outcome, death, and symptomatic intracerebral hemorrhage (sICH), were collected from the prospective registries. Functional outcome and death were assessed based on the mRS score at 3 months after stroke onset. A favorable outcome was defined as mRS score of 0–2. The ICH was assessed on CT or MRI scans obtained 24 ± 6 h after EVT. If the patient's neurological status worsened, the CT or MRI scans were obtained anytime to evaluate ICH. The ICH was defined as symptomatic if the patient's NIHSS score increased ≥4.

### Statistical Analyses

Based on the different types of endovascular modalities used to recanalize ICAS-LVO, endovascular strategies were classified into four groups: (1) EVT performed only with MT modalities (*MT-alone group*); (2) among ISMs, RS performed after MT failure (*MT-RS group*); (3) GPI infused after MT failure (*MT-GPI group*); and (4) both RS and GPI infusion performed after MT failure (*MT-RS*+*GPI group*).

First, demographics, risk factors for stroke, procedural details and outcomes, follow-up arterial patency, and clinical outcomes, including functional outcome, death, and sICH, were compared among the groups. Analysis of variance, Kruskal–Wallis test, χ^2^ test, and Fisher exact test were used as appropriate. Second, to evaluate whether endovascular strategy was independently associated with functional outcome, a multivariable binary logistic regression analysis was performed for favorable outcome. Variables with potential association (*p* < 0.2 in univariable analyses) were entered into the multivariable model. For the univariable analyses, Student *t*-test, Mann–Whitney *U*-test, χ^2^ test, and Fisher exact test were used as appropriate.

A *p* < 0.05 was considered statistically significant for 95% confidence interval (CI). All statistical analyses were performed using SPSS software (version 23; IBM, Armonk, NY, USA).

## Results

**Figure 1:**
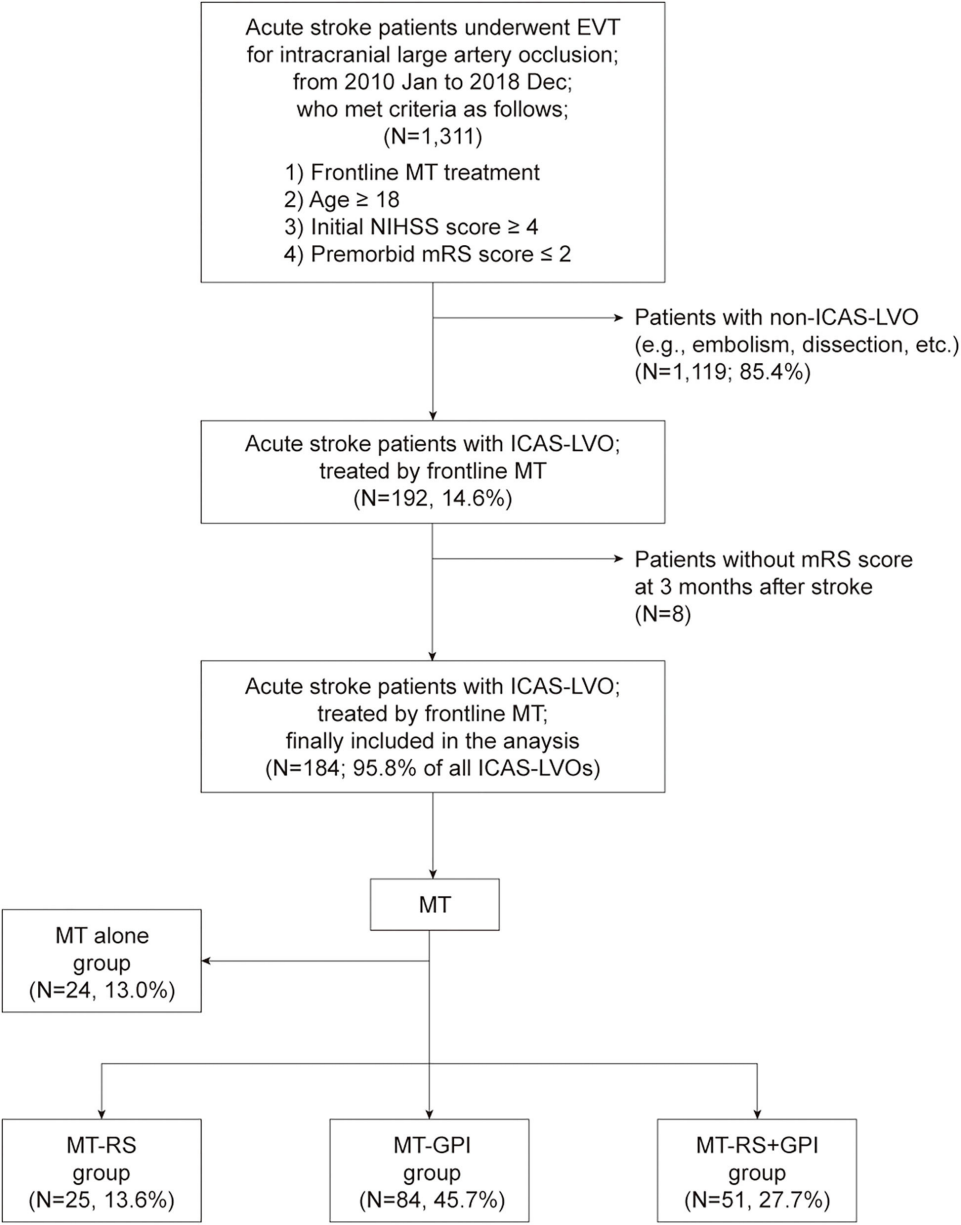
Patient selection flow and grouping. EVT, endovascular treatment; MT, mechanical thrombectomy; NIHSS, National Institutes of Health Stroke Scale; mRS, modified Rankin Scale; ICAS-LVO, intracranial atherosclerosis-related large-vessel occlusion; MT-RS, rescue stenting after mechanical thrombectomy failure; MT-GPI, glycoprotein IIb/IIIa infusion after mechanical thrombectomy failure; MT-RS+GPI, rescue stenting with glycoprotein IIb/IIIa infusion after mechanical thrombectomy failure.

Among the 1,311 acute stroke patients who met the inclusion criteria for this study, 192 (14.6%) had ICAS-LVO. After excluding patients without a 3-month mRS score, 184 patients (95.8% of all patients with ICAS-LVO; mean age, 67.9 ± 14.0 years; male, 57.6%) were included in the final analysis (Figure 1). Twenty-four patients (13.0%) were treated only with MT modality (MT-alone group). ISMs were used in 160 patients (87.0%): 25 (13.6%) treated with RS (MT-RS group), 84 (45.7%) with GPI infusion (MT-GPI group), and 51 (27.7%) with both RS and GPI infusion (MT-RS+GPI group). Age, initial NIHSS score, and time from stroke onset to puncture differed among the groups (Table 1). SRT was the predominant frontline MT modality in all groups, and CAT was used as the frontline modality in ~30% of patients in MT alone and MT-RS groups.

**Table 1:** Clinical and procedural characteristics and outcomes based on endovascular modality for intracranial atherosclerosis-related large-vessel occlusion.

	**MT alone (*n* = 24)**	**MT-RS (*n* = 25)**	**MT-GPI (*n* = 84)**	**MT-RS+GPI (*n* = 51)**	***p*-Value**
**Characteristics**					
Age, years	74.6 (± 11.9)	69.7 (± 15.6)	65.8 (± 13.2)	67.4 (± 14.6)	0.045
Male	11 (45.8)	16 (64.0)	52 (61.9)	27 (52.9)	0.412
Hypertension	18 (75.0)	14 (56.0)	61 (72.6)	34 (66.7)	0.388
Diabetes	8 (33.3)	8 (32.0)	24 (28.6)	13 (25.5)	0.887
Hypercholesterolemia	7 (29.2)	6 (24.0)	25 (29.8)	12 (23.5)	0.849
Smoking	5 (20.8)	6 (24.0)	30 (35.7)	14 (27.5)	0.414
Coronary artery disease	1 (4.2)	1 (4.0)	5 (6.0)	4 (7.8)	0.890
Atrial fibrillation	3 (12.5)	3 (12.0)	10 (11.9)	5 (9.8)	0.680
Initial NIHSS score	15.5 [11.8; 19.3]	17.0 [15.5; 18.0]	12.0 [7.0; 15.0]	15.5 [12.5; 17.0]	<0.001
Occlusion site					0.403
Internal carotid artery	9 (37.5)	5 (20.0)	18 (21.4)	13 (25.5)	
Middle cerebral artery	15 (62.5)	20 (80.0)	66 (78.6)	38 (74.5)	
ASPECTS	7.5 [6, 9]	8 [7, 9]	8 [7,8]	8 [7,9]	0.174
Use of IV tPA	11 (45.8)	5 (20.0)	25 (29.8)	22 (43.1)	0.101
Frontline MT modality					0.003
Stent retriever	17 (70.8)	18 (72.0)	78 (92.9)	47 (92.2)	
Contact aspiration	7 (29.2)	7 (28.0)	6 (7.1)	4 (7.8)	
No. of MT passes	2.9 (± 1.5)	3.2 (± 2.0)	2.6 (± 1.7)	2.4 (± 1.1)	0.186
Time of onset to puncture, min	290 [183; 412]	259 [222; 375]	488 [211; 922]	290 [240; 444]	0.005
Total procedure time, min	66 [53; 93]	95 [90; 99]	133 [83; 156]	144 [104; 166]	0.035
Post-procedural antithrombotics					<0.001
None	18 (75.0)	17 (68.0)	10 (11.9)	7 (13.7)	
Oral antiplatelets immediately after procedure	6 (25.0)	7 (28.0)	1 (1.2)	0 (0.0)	
IV GPI infusion followed by oral antiplatelets	0 (0.0)	1 (4.0)	73 (86.9)	44 (86.3)	
**Outcome**					
Recanalization					
Successful recanalization	18 (75.0)	20 (80.0)	80 (95.2)	50 (98.0)	0.001
Patent artery on follow-up	17 (70.8)	14 (56.0)	70 (83.3)	50 (98.0)	<0.001
Favorable outcome	7 (29.2)	11 (44.0)	55 (65.5)	43 (84.3)	<0.001
Symptomatic ICH	2 (8.3)	2 (8.0)	2 (2.4)	1 (2.0)	0.323
Mortality	3 (12.5)	5 (20.0)	2 (2.4)	1 (2.0)	0.003

*MT, mechanical thrombectomy; MT-RS, rescue stenting after mechanical thrombectomy failure; MT-GPI, glycoprotein IIb/IIIa infusion after mechanical thrombectomy failure; MT-RS+GPI, rescue stenting with glycoprotein IIb/IIIa infusion after mechanical thrombectomy failure; ASPECTS, Alberta Stroke Program Early Computed Tomography Score; IV tPA, intravenous tissue plasminogen activator; ICH, intracerebral hemorrhage; NIHSS, National Institutes of Health Stroke Scale*.

*Values in parentheses represent the standard deviation or the number of patients (%). Brackets represent first and third quartiles*.

### Recanalization Results Based on Endovascular Modality

A successful recanalization was achieved in 168 patients [91.3% (168 of 184)]. MT was successful in only 9.8% (18 of 184) of all ICAS-LVO patients as a frontline modality (Table 1). Among the remaining 166 patients who did not experience a successful recanalization with the frontline MT treatment, 160 were further treated with ISM. Rescue treatment with ISM was effective in 150 patients [150 of 160 (93.8%); overall recanalization efficiency of ISM after MT failure]. Recanalization efficiency in the MT-RS group (80.0%) was significantly lower than in the MT-GPI (95.2%, *p* = 0.028) and MT-RS+GPI groups (98.0%, *p* = 0.013).

### Outcomes Based on Endovascular Modality

**Figure 2:**
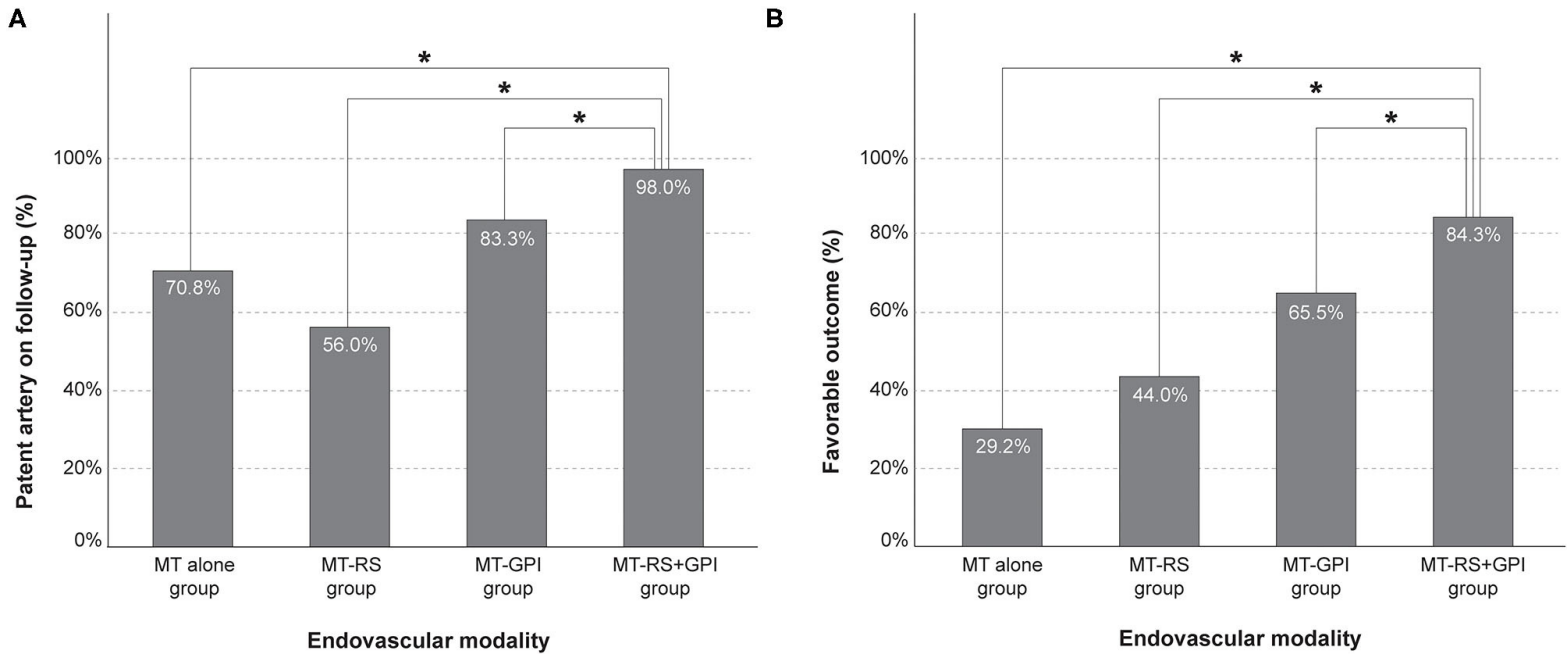
Frequency of **(A)** patent artery on follow-up and **(B)** favorable outcome based on endovascular modality. MT-RS, rescue stenting after mechanical thrombectomy failure; MT-GPI, glycoprotein IIb/IIIa infusion after mechanical thrombectomy failure; MT-RS+GPI, rescue stenting with glycoprotein IIb/IIIa infusion after mechanical thrombectomy failure. *Significantly different between groups.

On the follow-up examination, 89.9% of recanalized arteries (151 of 168) were patent. The frequency of patent arteries on follow-up was different based on endovascular modality (*p* < 0.001; Table 1) and most frequent in the MT-RS+GPI group (98.0%). Patent arteries in the MT-RS+GPI group were significantly more frequent than in the MT alone (70.8%, *p* = 0.001), MT-RS (56.0%, *p* < 0.001), and MT-GPI groups (83.3%, *p* = 0.009; Figure 2A). The frequency of patent arteries on follow-up in the MT-RS group was significantly lower than in the MT-GPI group (*p* = 0.004) and not significantly different from the MT-alone group.

Functional outcome was also different based on endovascular modality (*p* < 0.001; Table 1). Favorable outcome was observed most in the MT-RS+GPI group (84.3%) and the least in the MT-alone group (29.2%). Furthermore, favorable outcome in the MT-RS+GPI group was significantly more frequent than in MT alone (*p* < 0.001), MT-RS (44.0%, *p* < 0.001), and MT-GPI groups (65.5%, *p* = 0.017; Figure 2B). Favorable outcome in the MT-GPI group was significantly more frequent than in the MT-alone group (*p* = 0.002). However, functional outcomes in the MT-RS group were not significantly different from the MT-alone group. Mortality was different based on endovascular modality, which was highest in the MT-RS group. The sICH did not differ among the groups.

The type of endovascular modality used for treating ICAS-LVO was also an independent factor for favorable outcome compared with using only the MT modality. Multivariable analysis showed the MT-RS+GPI modality [odds ratio (OR), 20.4; 95% CI, 1.97–211.4; *p* = 0.012] remained an independent factor for favorable outcomes, in addition to younger age, low initial NIHSS score, lower number of MT device passes, successful recanalization, patent artery on follow-up, and use of intravenous infusion of GPI followed by oral antiplatelets (Table 2).

**Table 2:** Factors associated with favorable outcome.

	**Univariable**			**Multivariable**	
	**Favorable outcome (*n* = 116)**	**Unfavorable outcome (*n* = 68)**	***p*-value**	Odds ratio^*^ (95% CI)	***p*-value**
Age, years	64.2 (± 14.8)	74.1 (± 9.9)	<0.001	0.95 (0.91–0.99)	0.018
Male	76 (65.5)	30 (44.1)	0.005	0.79 (0.30–2.08)	0.636
Hypertension	78 (67.2)	49 (72.1)	0.514		
Diabetes	28 (24.1)	25 (36.8)	0.091	0.69 (0.24–1.98)	0.488
Hypercholesterolemia	28 (24.1)	22 (32.4)	0.235		
Smoking	44 (37.9)	11 (16.2)	0.002	3.19 (0.97–10.5)	0.056
Coronary artery disease	5 (4.3)	6 (8.8)	0.334		
Atrial fibrillation	10 (8.6)	11 (16.2)	0.150	0.45 (0.11–1.88)	0.275
Initial NIHSS score	13.0 [8.0; 16.0]	16.0 [12.0; 20.0]	<0.001	0.90 (0.82–0.98)	0.017
Occlusion site			0.903		
Internal carotid artery	28 (24.1)	17 (25.0)			
Middle cerebral artery	88 (75.9)	51 (75.0)			
Use of IV tPA	45 (38.8)	18 (26.5)	0.108	1.69 (0.62–4.66)	0.308
Time of onset to puncture, min	320 [239; 609]	290 [202; 710]	0.631		
Total procedure time, min	94 [60; 157]	104 [84; 147]	0.781		
Frontline MT modality			0.024		
Stent retriever	106 (91.4)	54 (79.4)		Reference	
Contact aspiration	10 (8.6)	14 (20.6)		0.71 (0.15–3.30)	0.667
No. of MT passes	2.2 (± 1.0)	3.5 (± 2.1)	<0.001	0.47 (0.31–0.71)	<0.001
Endovascular modalities			<0.001		
MT alone	7 (6.0)	17 (25.0)		Reference	
MT-RS	11 (9.5)	14 (20.6)		1.41 (0.07–27.9)	0.822
MT-GPI	55 (47.4)	29 (42.6)		3.21 (0.44–23.5)	0.250
MT-RS+GPI	43 (37.1)	8 (11.8)		20.4 (1.97–211.4)	0.012
Successful recanalization	113 (97.4)	55 (80.9)	<0.001	8.48 (1.01–71.8)	0.049
Patent artery on follow-up	109 (94.0)	42 (61.8)	<0.001	14.1 (2.05–97.4)	0.007
Postprocedural antithrombotics			<0.001		
None	18 (15.5)	34 (50.0)		Reference	
Oral antiplatelets immediately after procedure	8 (6.9)	6 (8.8)		1.62 (0.26–9.96)	0.603
IV GPI infusion followed by oral antiplatelets	90 (77.6)	28 (41.2)		22.8 (1.09–475.9)	0.044

*IV tPA, intravenous tissue plasminogen activator; MT, mechanical thrombectomy; MT-RS, rescue stenting after mechanical thrombectomy failure; MT-GPI, glycoprotein IIb/IIIa infusion after mechanical thrombectomy failure; MT-RS+GPI, rescue stenting with glycoprotein IIb/IIIa infusion after mechanical thrombectomy failure; IV GPI, intravenous glycoprotein IIb/IIIa inhibitor; NIHSS, National Institutes of Health Stroke Scale*.

*Values in parentheses represent the standard deviation or the number of patients (%). Brackets represent first and third quartiles*.

* *Odds ratio for favorable outcome*.

## Discussion

In this study, frontline MT was not very effective in ICAS-LVO and resulted in <10% of successful recanalization rate. For patients who experienced failed frontline MT modality, successful recanalization was achieved in ~94% of patients treated with ISM. Recanalization efficiency using a combination of RS and GPI infusion was relatively higher than with other ISMs. Among ISMs, a combination of RS and GPI infusion resulted in significantly more patent arteries on follow-up and significantly more favorable outcomes, which was an independent factor for favorable outcome.

In several previous reports, conventional MT modalities were shown to be ineffective for ICAS-LVO; thus, alternative or special strategies for ICAS-LVO are necessary for better EVT outcomes (9–12, 20). Based on a small number of retrospective studies in which the procedural details and outcomes were analyzed, most rescue modalities for ICAS-LVO included immediate intracranial stenting, percutaneous balloon angioplasty, GPI infusion, and combinations of the modalities (referred to as ISMs) (3–5, 13, 14, 21–23). The necessity of ISMs in EVT for ICAS-LVO is generally recognized; however, unfortunately, the optimal ISM remains unknown. In clinical practice, one type of ISM should be chosen after frontline MT device fails. To make the best decision, many factors should be considered including which ISM provides a greater possibility to obtain a successful recanalization, whether the recanalized target artery will be well-maintained (or patent) after the endovascular procedure, whether the chosen ISM will cause intracranial hemorrhage, and whether patient's clinical outcome will be guaranteed when the specific ISM is used. If more information regarding these factors is known, the decision would likely be easier and more rational.

From a strategic viewpoint, the results from this study indicated that RS alone was not appropriate as the rescue endovascular modality. RS alone performed after frontline MT failure (MT-RS group) was not significantly more beneficial regarding arterial patency on follow-up and functional outcome. In addition, patients in the MT-RS group showed the lowest recanalization efficiency among ISMs, and mortality was rather high. Conversely, several beneficial effects were observed when using a combination of RS and GPI infusion after MT failure (MT-RS+GPI group) such as recanalization efficiency, follow-up arterial patency, and favorable outcome. Approximately 98% of patients had a successful recanalization with the combination of RS and GPI infusion. Patent artery on follow-up and favorable outcome were significantly more frequent in the MT-RS+GPI group. Furthermore, use of a combination of RS and IA GPI infusion was an independent factor for favorable outcome. Unlike common anxiety regarding hemorrhagic risk, GPI infusion was not associated with sICH development, which is in agreement with the results from previous studies (22–25). Because successful recanalization and patent artery on follow-up were significant factors affecting favorable outcome, a combination of RS and GPI infusion might result in a better patient clinical outcome due to higher recanalization efficiency and more patent arteries observed on follow-up. In fact, delayed reocclusion after EVT was highly associated with poor functional outcome, which was represented by a quite low OR for favorable outcome (0.035; 95% CI, 0.005–0.243) (26). According to a recent large registry, worsening of arterial patency was significantly associated with all kinds of negative clinical outcomes including early neurological deterioration, short- and long-term mortality, and poor functional status (OR, 5.37; 95% CI, 2.70–8.49) (27).

Although favorable outcome in the MT-GPI group was not comparable with the MT-RS+GPI group (the absolute difference was ~20%), patients in the MT-GPI group experienced relatively good recanalization efficiency and follow-up arterial patency. On multivariable analysis, the OR in the MT-GPI group showed a tendency for a favorable outcome, although not significantly. Compared with the MT-RS group, patients in the MT-GPI group had better recanalization efficiency, follow-up arterial patency, and more favorable outcome. Therefore, GPI infusion may play a role in MT failure. To examine the role of GPI infusion after MT failure, further prospective studies are necessary.

The MT-RS+GPI group showed a much higher rate of favorable outcome than expected in general. Although this study did not focus on the specific factors for the remarkable clinical outcome in the MT-RS+GPI group, we think that patent artery on follow-up, active and immediate administration of postprocedural antithrombotics, or a kind of bias such as the selection of a smaller lesion for further use of ISM might affect the outstanding clinical outcome. However, interpretation should be cautious as the rate of favorable outcome in the MT-RS+GPI group was not statistically different from that in the MT-GPI group in multiple comparisons.

This study had several limitations. First, because of the retrospective nature of this study, procedural decisions were not regulated under a specific protocol. The timing of frontline MT failure was determined according to operators' best judgment. Thus, successful recanalization using the frontline MT procedure might have been underestimated. However, the mean number of MT passes in all groups was not significantly different. More importantly, the main focus in this study was rescue endovascular modalities specific to ICAS-LVO, not MT failure. Second, the choice of ISM may be biased. Hemorrhagic risk is the most common consideration when using ISM. Many physicians are concerned that GPI infusion or postprocedural antithrombotics after emergent stenting elicit intracranial bleeding. Therefore, the use of ISM may be biased in patients with less risk of hemorrhagic complications. A few clinical factors relevant to hemorrhagic risk, such as a lesion size, might also affect the choice of ISM. However, lesion sizes representing initial ASPECTS were not significantly different between groups in this study. Moreover, the focus of this study was on the type of ISM and not on whether to use ISM itself; all types of ISM used in this study were thought equivalent, at least when hemorrhage was considered. Third, the sequence of GPI infusion and RS was not specified in this study. One might first consider using an easier or pharmacologic one and thus conduct an escalating method—GPI infusion first, then RS if GPI fails. In other cases, GPI can be infused after RS. However, this study focused only on the type of further ISM, but not on its sequence. Thus, we did not distinguish the different sequences of combination. A prospective study is necessary to verify the treatment effectiveness according to its sequence. Fourth, this study was conducted in an Asian country, where ICAS is more prevalent than in Western countries. However, ICAS is also an important issue for Hispanic and African populations. Furthermore, overcoming refractoriness to modern MT techniques should be discussed regardless of patient's race. Consequently, more specific improvements to the endovascular strategy for ICAS-LVO are necessary. Evaluating and comparing procedural and clinical outcomes based on the types of rescue modalities would be of great importance in the field of EVT for acute stroke.

## Conclusions

Rescue endovascular strategy after MT failure was significantly associated with procedural and clinical outcomes in acute stroke caused by ICAS-LVO. Use of a combination of RS and GPI infusion showed the highest rate of recanalization efficiency, patent arteries on follow-up, and favorable outcome. A combination of RS and GPI infusion might be an optimal rescue modality when frontline MT fails in EVT of ICAS-LVO.

## Data Availability Statement

The raw data supporting the conclusions of this article will be made available by the authors, without undue reservation.

## Ethics Statement

The studies involving human participants were reviewed and approved by Institutional Review Boards of all participating centers. Written informed consent for participation was not required for this study in accordance with the national legislation and the institutional requirements.

## Author Contributions

BMK established the study idea and analyzed the study data. J-HB and CJ contributed to acquisition of the study data, interpretation of the analysis, and draft of this manuscript. BMK, JHH, DJK, HSN, YDK, EHL, J-HK, JYK, and JHK contributed to acquisition of the study data, interpretation of the analysis, and critical revisions to the manuscript. All authors contributed to the article and approved the submitted version.

## Conflict of Interest

The authors declare that the research was conducted in the absence of any commercial or financial relationships that could be construed as a potential conflict of interest.
